# Virtual Reality Training Using Nintendo Wii Games for Patients With Stroke: Randomized Controlled Trial

**DOI:** 10.2196/29830

**Published:** 2022-06-13

**Authors:** Naveed Anwar, Hossein Karimi, Ashfaq Ahmad, Syed Amir Gilani, Kehkshan Khalid, Ahmed Sohaib Aslam, Asif Hanif

**Affiliations:** 1 University Institute of Physical Therapy University of Lahore Lahore Pakistan; 2 Department of Physical Therapy Nur International University Lahore Pakistan; 3 Department of Physical Therapy Avicenna Medical College Lahore Pakistan; 4 Department of Physical Therapy Kanaan Healthcare Center Lahore Pakistan

**Keywords:** stroke, virtual reality, Fugl-Meyer score, rehabilitation, training, physical therapy, therapy, balance, function, randomized controlled trial

## Abstract

**Background:**

Stroke is a leading cause of disability. It is difficult to devise an optimal rehabilitation plan once stroke survivors are back home. Conventional rehabilitative therapies are extensively used in patients with stroke to recover motor functioning and disability, but these are arduous and expensive. Virtual reality (VR) video games inspire patients to get involved in their therapeutic exercise routine in a fun way. VR in the form of games provides a fruitful, secure, and challenging learning environment for motor control and neural plasticity development in rehabilitation. The effects of upper limb sensorimotor functioning and balance are the main focus of this trial.

**Objective:**

The aim of this study is to compare the effects of VR training and routine physical therapy on balance and upper extremity sensorimotor function in patients with stroke.

**Methods:**

It was a single assessor-blinded randomized clinical trial. A total of 74 participants with their first chronic stroke were included and rehabilitated in a clinical setting. The lottery method was used to randomly assign patients to either the VR group (n=37) or the routine physical therapy group (n=37). The VR group received a 1-hour session of VR training for 3 weekdays over 6 weeks, and the routine physical therapy group received different stretching and strengthening exercises. The outcome measuring tools were the Berg Balance Scale for balance and the Fugl-Meyer Assessment (upper extremity) scale for sensorimotor, joint pain, and range assessment. The assessment was done at the start of treatment and after the 6 weeks of intervention. Data analysis was done using SPSS 22.

**Results:**

The trial was completed by 68 patients. A significant difference between the two groups was found in the Berg Balance Scale score (*P*<.001), Fugl-Meyer Assessment for motor function (*P*=.03), and Fugl-Meyer Assessment for joint pain and joint range (*P*<.001); however, no significant difference (*P*=.19) in the Fugl-Meyer Assessment for upper extremity sensation was noted.

**Conclusions:**

VR training is helpful for improving balance and function of the upper extremities in the routine life of patients with stroke; although, it was not found to be better than conventional training in improving upper limb sensation. VR training can be a better option in a rehabilitation plan designed to increase functional capability.

**Trial Registration:**

Iranian Registry of Clinical Trials RCT20190715044216N1; https://www.irct.ir/user/trial/40898/view

## Introduction

Stroke is a leading cause of disability, and it is difficult to devise an optimal rehabilitation plan for patients with stroke once they are discharged from the hospital [1]. Almost 85% of patients with stroke have hemiparesis after stroke while 55% to 75% of stroke survivors have motor dysfunction. South Asian people (people of India, Pakistan, Sri Lanka, Bangladesh, Nepal, and Bhutan) have a higher risk of stroke because of compromised cardiac and metabolic rate [2,3]. Treatment for stroke is initiated with drugs [4], and surgery might be another option to repair any constriction or narrowing of blood vessels [5,6]. Patient rehabilitation is an important part of treatment. The main purpose of rehabilitation is to improve the quality of life for patients with stroke and make them independent [7,8]. Conventional rehabilitative therapies are extensively used to help patients with stroke recover motor functioning and disability. However, the application of conventional techniques is arduous and expensive, and requires transportation of patients to tertiary care hospitals especially in countries like Pakistan where hospitals are less in number. Virtual reality (VR) training in the form of games [9] provides a fruitful, secure, and challenging learning environment for motor control and neural plasticity development after stroke. VR video games inspire patients to get involved in their therapeutic exercise routine in a fun way [10]. Depending on the remodeling and reorganization of brain function, previous studies found that VR can be a great alternative for quick functional recovery after stroke [11]. Mirror neurons in the cortex can be activated through observational learning by VR training. Participants who received sensory input in VR training were also more likely to learn the desired motor behavior [12]. The feedback can help to promote the development of use-dependent cortical plasticity, which could lead to improved motor control. Furthermore, the functional improvement induced by VR training could significantly boost participants’ confidence and self-efficacy in a new environment. Moreover, another advantage of VR is that it can greatly save on labor and cost of patients [13].

Stroke is seen to be more prevalent in countries like Pakistan, as the people are more inclined toward using local drugs like naswar, pipe smoking, and beetle leaf chewing (paan). Thus, there is higher incidence of stroke in middle-aged populations (<45 years) [14-16]. The study aimed for a younger population with stroke and found cost-effective treatment protocols at the same time. The unique needs of young people with stroke and the promising opportunity provided by a low-cost serious game would be a beneficial addition in treatment strategy. There is inadequate evidence in the literature to generalize effects on upper limb sensorimotor function and gait through commercial gaming in young patients with stroke. Studies on effectiveness of VR programs in comparison to traditional methods on functional-motor improvement of an upper limb are needed in low-resource countries to reduce cost and time through target-oriented interventions. This study was conducted to compare the effects of VR training and routine physical therapy on balance and function of upper limbs in patients with stroke from the lower- and middle-class populations. This study is conducted to accept or reject the hypothesis that VR has a significantly better effect on balance and upper limb function in patients with stroke.

## Methods

### Study Design and Participants

This study was a single assessor-blinded randomized clinical trial. Participants were recruited by convenient sampling at Kanaan Physiotherapy & Spine Clinic, Lahore, Pakistan, from September 2018 to December 2020. Diagnosed patients with subacute and chronic stroke were included in the study. The inclusion criteria were patients aged between 40 to 60 years irrespective of gender; unilateral involvement of extremity and the first episode of stroke was either hemorrhagic or ischemic in origin evident by computed tomography scan or magnetic resonance imaging [17]; at least a score of 2 or more on the medical research council scale; and patient is stable, alert, and able to follow the instructions of physical therapists. Patients with ischemic heart disease with unstable angina, history of seizures, Parkinson disease, severe aphasia that can limit participation or feedback, cognitive mental condition that can interfere with comprehension of commands, any systematic disease, BMI, or poor speech were excluded from the study. Written informed consent was obtained from each participant prior to data collection. The participants were randomly assigned to two groups by lottery method: VR (n=37) and routine physical therapy (n=37) groups [16].

### Outcome Measures

All values were measured before and after the 6 weeks of intervention. The Berg Balance Scale (BBS) was used to assess balance. It is a 14-item list with each item consisting of a five-point ordinal scale ranging from 0 to 4, with 0 indicating the lowest level of function and 4 the highest level of function. A score of 56 indicates functional balance. A score of <45 indicates individuals may be at greater risk of falling. The Fugl-Meyer Assessment (FMA) tool for upper extremities (UEs) was used to assess sensorimotor function, joint range, and pain. The motor section of the FMA-UE has 33 points that evaluates aspects of movement, reflex, coordination, and speed. Each domain contains multiple items, each scored on a 3-point ordinal scale (0=cannot perform, 1=performs partially, 2=performs fully). Sensation has 6 points, while joint range and pain have 12 points each. Scoring is based on direct observation of performance. FMA-UE is a valid and reliable tool that measures the function of upper limbs, wrists, and hands while the BBS is also a valid and reliable tool to assess balance [18,19].

### Interventions

#### Virtual Reality Training (Group I)

Wii comes with a console, adapter, infrared sensor bar, 2 wireless nunchucks, remote with wrist straps, sensor bar, Wii balance board, and Wii Sports kit. The Wii Sports (tennis and boxing), Wii balance board, and Wii Cooking Mama games were the main games used. Depending upon the participant’s ability, the training complexity and intensity were increased by higher levels in the game. The therapist stood behind the participant for protection and support, and the participant was able to grab the handrail if they needed to avoid falling [20]. Patients in this group had a 1-hour session 3 days a week for a period of 6 weeks.

#### Routine Physical Therapy (Group II)

These include stretching exercises for tight muscles (eg, shoulder, elbow, and wrist flexors). The strengthening program includes exercises for weak extensor muscles and balance training, and coordination exercises to improve motor control and deficit. Each muscle group was targeted for strengthening exercises in upper limbs. Manual resistance was applied and increased according to the patient’s condition. The patients in this group got 1-hour sessions, 3 days a week for a period of 6 weeks.

### Sample Size

**Figure 1:**
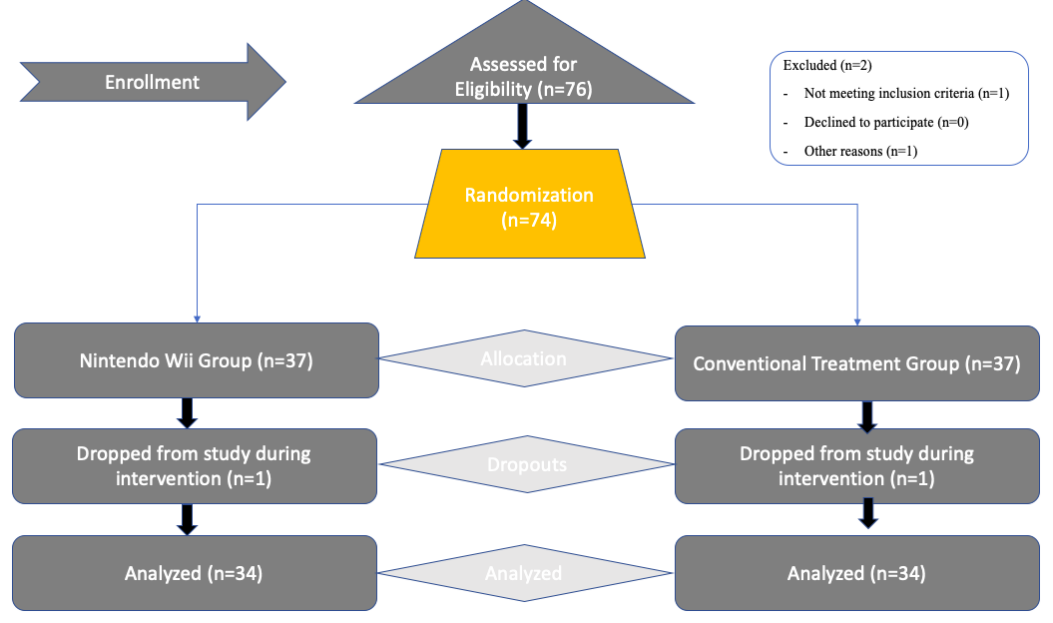
CONSORT (Consolidated Standards of Reporting Trials) flow diagram.

The sample size was calculated by sample size software [21,22]. The sampling technique used was convenient sampling with a statistical power of 80% and ∂=0.5. Recruitment of 76 participants was done with an expected dropout of (10%) during the intervention. One patient was excluded for not meeting the criteria, and 1 left due to personal reasons. During the intervention, 3 patients from each group were lost to follow-up as shown in Figure 1.

### Ethical Approval

The study protocol was approved by the Ethical Committee of the University of Lahore (approval IRB-UOL-FAHS/373-III/2018).

### Randomization

Eligible participants were initially screened by a research assistant. All participants were asked to sign the informed consent. Subsequently, the participants were assessed by a blinded independent assessor at baseline and were randomized. The randomization was done by lottery method. Patients were given the allotted intervention by a trained physical therapist. After 6 weeks of the intervention, another assessment was done by a blinded independent assessor.

### Statistical Analysis

SPSS 22.0 (IBM Corp) was used for the statistical analysis. Descriptive statistics were applied for all outcome measures. To check the normality of data, the Shapiro-Wilk test was performed. Data were found to be normally distributed, so parametric tests were applied. An independent *t* test was used to compare data between the groups, while a paired *t* test was used for analysis within the group. Statistical significance was set at *P*<.05.

## Results

The data were normally distributed. The mean age of the VR training group was 51.56 (SD 7.199) years, while the mean age for the routine physical therapy group was 51.35 (SD 5.783) years. The mean height of the VR training group was 1.68 (SD 0.11) meters, while the mean height for the routine physical therapy group was 1.69 (SD 0.09) meters. The mean weight of the VR training group was 86.50 (SD 11.41) Kg, while the mean weight for routine physical therapy was 86.35 (SD 11.7) Kg as shown in Table 1.

There was statistically significant differences between posttreatment BBS values of the 2 groups with *P*<.001. The balance increased to a greater extent post treatment for the VR training group with a mean value of 36.62 (SD 7.76) as compared to the routine physical therapy group (mean 26.94, SD 6.46) as shown in Table 2. The mean of the FMA score for motor function in the VR training group was 49.71 (SD 10.03) as compared to 33.47 (SD 11.07) in the routine physical therapy group, which was clinically more significant in the VR group. There was no significant difference in the FMA score for sensation across the 2 groups with *P*=.19. There was significant difference in the FMA for joint pain and joint range across the 2 groups with *P*<.001 as shown in Table 2. A paired sample *t* test was used to compare the values within each treatment group.

**Table 1:** Demographic characteristics (N=68).

Study groups	Patients, n	Value, mean (SD)
Age (years; virtual reality training)	34	51.56 (7.199)
Age (years; routine physical therapy)	34	51.35 (5.783)
Weight (kg; virtual reality training)	34	86.50 (11.41)
Weight (kg; routine physical therapy)	34	86.35 (11.70)
Height (m; virtual reality training)	34	1.68 (0.11)
Height (m; routine physical therapy)	34	1.69 (0.09)
BMI (kg/m^2^; virtual reality training)	34	30.66 (4.24)
BMI (kg/m^2^; routine physical therapy)	34	30.60 (4.67)

**Table 2:** Pre- and postintervention results for virtual reality and routine physical therapy treatment: t test between group statistics (N=68).

Berg Balance Scale and FMA^a^ for upper extremity			Treatment group					*P* value
			Virtual reality training, mean (SD)		Routine physical therapy, mean (SD)			
Berg Balance Scale score								<.001
	Pre	18.38 (5.19)		19.68 (5.23)				
	Post	36.62 (7.76)		26.94 (6.46)				
FMA for motor function								<.001
	Pre	19.97 (5.45)		21.03 (7.24)				
	Post	49.71 (10.03)		33.47 (11.07)				
FMA for sensation								.19
	Pre	7.62 (2.15)		7.24 (2.41)				
	Post	8.44 (1.99)		8.44 (2.5)				
FMA for joint range								<.001
	Pre	12.82 (4.26)		11.88 (3.88)				
	Post	18.18 (3.71)		14.56 (4.13)				
FMA for joint pain								<.001
	Pre	12.47 (3.79)		11.65 (3.16)				
	Post	19.62 (2.86)		14.62 (4.00)				

^a^FMA: Fugl-Meyer Assessment.

## Discussion

### Principal Findings

The aim of the study is to compare the effects of two techniques, VR training and routine physical therapy, on balance and function of upper extremities in patients with stroke. Virtual training is a modern technique that has more interest now because of its affordability and that patients can easily use it at home as well. VR training improved balance, pain, range of motion, and motor function of the upper extremities. However, there was no significant effect on sensation.

This study had a significant result in the improvement of the balance during group analysis. Posttreatment balance in the VR group had a mean value of 36.62 (SD 7.76), as compared to the routine physical therapy group (mean 26.94, SD 6.46). Similar results were seen in a study conducted by Jeon et al [23] that suggested that VR in combination with balance training had significant improvement in patients with stroke as compared to the VR alone. Another study conducted by Aramaki et al [24] showed that VR training improved balance and upper limb function, and improved the quality of life in patients with stroke, who also reported that VR is more effective in improving dynamic balance as compared to conventional treatment. Specifically, balance in walking was the most underperformed activity of patients with stroke, so improvement in it would be a substantial help for them. Most of the patients considered playing games as a fun activity in their leisure time. Routine physical therapy was found to be somewhat boring for patients and less attractive [11]. The results of this study will help therapists to use VR for the rehabilitation of patients with stroke. A patient tries to use his muscles, and thus, coordination with the brain improves. Most of the postural muscles are involved in an upright position. On the contrary, routine physical therapy is somewhat passive, and thus improvement is slow. Postural muscles should be targeted in treatment protocols. Sometimes, stroke directly affects the patient’s ability to manage the surrounding environment. So, it is difficult to balance yourself when you are unsure about your position in the surroundings [25-27]. This study proved that VR improves functional independence of patients after stroke.

In this study, both groups experienced positive improvements in the FMA on the motor function level. The study’s posttreatment results of motor function in patients with stroke had a mean value of 49.71 (SD 10.03) in the VR group, while in the routine physical therapy group, where the mean value was 33.47 (SD 11.07) after treatment, the score was substantially higher than the baseline, suggesting a clinically meaningful increase in the VR training community relative to the routine physical therapy group. A study conducted by Maier et al [28] investigated whether VR training was beneficial for upper limb motor recovery as compared to conventional therapy. A review conducted by Levin and Demers [12] also concluded that VR training has significant results in improving balance and motor function of upper limbs in stroke rehabilitation.

Motor function in upper limbs improves during virtual training because this system provides extra spatial transformation and uncoupled eye-hand movements; it also enhances movement control and creates an entertaining environment, further motivating patients. Repeated motions improve motor learning and patient’s functional and anatomical reorganization [29].

The study showed nonsignificant results of sensation by using VR in patients with stroke, the VR group posttreatment mean value was 8.44 (SD 1.99) as compared to the routine physical therapy group (mean 8.44, SD 2.5). A study conducted by Yeh et al [17] showed that the VR system helps promote functional movements and motor control like pinching and grasping activity, with significant results, but our sensory control patients strongly intended to continue it for a long time. Another study conducted by Serrada et al [30] highlights the sensory training, but limited evidence was available on the sensory impairment of patients with stroke. Further evaluation is required to analyze the effectiveness for active sensory training. Vigorous methods and high-class research is needed to measure outcomes of sensory rehabilitation. This largely overlooked topic is an important component for stroke rehabilitation.

This study showed significant results in joint range of motion in the VR group. The VR group had a posttreatment mean value of 18.18 (SD 3.71) as compared to the routine physical therapy group (mean 14.56, SD 4.13). A study conducted by Aşkın et al [31] stated that kinetic VR training improved UE motor function and range of motion in patients with stroke. Another study conducted by Huang et al [32] suggested that VR training improves fine hand movements and active ranges of motion, and promotes coordination, which support the results from our study.

A study conducted by Choi and Paik [33] showed that a VR mobile game–based program for upper limb rehabilitation had significant results with conventional therapy, and it was a good tool for rehabilitation of the UE, indicating that the joint range is achieved in patients with stroke by VR training. Different visual and auditory sounds engage patients in activity, and patients spend more time in performing activities. This creates a desire of interaction, and at the end, a desired joint movement is achieved [33], similar to kinetic VR where shoulder flexion, abduction external rotation, and elbow extension significantly improved by repetitive training more than once a day.

The study results showed significant results in improving pain in the poststroke treatment VR group, which had a mean value of 19.62 (SD 2.86), as compared to routine physical therapy group, which had a mean of 14.62 (SD 4.00). A study conducted by Shahrbanian et al [34] showed that VR training helped in pain management as compared to conventional therapy. A study conducted by Powell and Simmonds [35] suggested that musculoskeletal pain caused activity limitation, but VR training could improve pain and enhance movement speed, which also supports our study results.

Another study conducted in 2014 by Triberti et al [36] stated that VR training improved pain. It is a distraction and analgesic tool, and creates an environment in which the patients immerse themselves in a 3D computer-generated environment. This pleasurable and colorful environment diverts attention from noxious stimuli. It provides a psychological effectiveness, reduces anxiety, and promotes positive emotions so patients feel relaxed and experience less pain [36].

VR has a great neurobiological impact on neuroplasticity that results in an improved volume of gray matter, improved cognitive efficiency, and higher electroencephalographic beta wave concentrations. Innovative brain-computer interfaces assist clinical applications of VR by allowing a direct effect on the electric activity caused by different cortical areas of the brain to ensure efficient control of connected gaming devices. Healthy people may use VR as a storytelling tool to rewrite their own stories as part of an integrative process of self-improvement and personal growth [37,38].

This study has some limitations, as VR training is a comparatively new technique in Pakistan, which is why patients faced difficulties with familiarity despite the fact that it is being used widely for rehabilitation in higher-income countries. Few outcome measures were used in the trial, so we suggest that more outcome measures be used in future trials. This trial did not have long-term follow-up assessments to check continuous effects. These limitations need to be addressed in future studies.

### Conclusions

In this study, VR training was an effective way to improve balance, sensorimotor function, joint range, and pain of the upper limbs. Routine physical therapy is beneficial, but VR training can be more target-oriented. This study has a lot of potential in the field of stroke rehabilitation, as it demonstrated that low-cost technologies can offer additional benefits to usual care. Moreover, further randomized controlled trials are required to find the effects of VR in different occupational performances in patients with stroke.

## Appendix

Multimedia Appendix 1 CONSORT-eHEALTH checklist (V 1.6.1).

## Abbreviations

BBS: Berg Balance Scale
FMA: Fugl-Meyer Assessment
UE: upper extremity
VR: virtual reality
